# Supplementary material for: Antibody targeting of E3 ubiquitin ligases for receptor degradation
Source: Nature. 2022 Sep 21;610(7930):182–9. doi: 10.1038/s41586-022-05235-6 (PMC9534761; doi:10.1038/s41586-022-05235-6)
Supplement: Supplementary file 1 — The Supplementary Information file includes three Supplementary Figures. Supplementary Figure 1 and 2 present uncropped blots of western blot panels presented in the main and Extended Data figures, respectively. Supplementary Figure 3 outlines the gating strategies used for flow cytometry analysis presented in the manuscript. [file 41586_2022_5235_MOESM1_ESM.pdf]

nature portfolio

https://doi.org/10.1038/s41586-022-05235-6

Supplementary information

Antibody targeting of E3 ubiquitin ligases for receptor degradation

In the format provided by the authors and unedited

Antibody targeting of E3 ubiquitin ligases for receptor degradation

Authors

Hadir Marei*[,1], Wen-Ting K. Tsai*[,2], Yee-Seir Kee*[,2], Karen Ruiz[1], Jieyan He[3], Chris Cox[4], Tao Sun[5], Sai Penikalapati[6], Pankaj Dwivedi[6], Meena Choi[6], David Kan[7], Pablo Saenz-Lopez[7], Kristel Dorighi[5], Pamela Zhang[2], Yvonne T. Kschonsak[1], Noelyn Kljavin[8], Dhara Armin[1], Ingrid Kim[2], Andrew G. Mancini[8], Thao Nguyen[8], Chunling Wang[1], Eric Janezic[3], Alexander Doan[3], Elaine Mai[3], Hongkang Xi[9], Chen Gu[10], Melanie Heinlein[8], Brian Biehs[8], Jia Wu[9], Isabelle Lehoux[11], Seth Harris[12], Laetitia Comps-Agrar[3], Dhaya Seshasayee[9], Frederic J. de Sauvage[8], Matthew Grimmer[1], Jing Li[3], Nicholas J. Agard[2], Felipe de Sousa e Melo[1]

Affiliations

Department of [1]Discovery Oncology, [2]Antibody Engineering, [3]Biochemical and cellular pharmacology, [4]Discovery Immunology, [5]Molecular Biology, [6]Microchemistry, Proteomics and Lipidomics, [7]Translational Oncology, [8]Molecular Oncology, [9]Antibody discovery, [10]Protein Chemistry, [11]Biomolecular Resources, [12]Structural Biology, Genentech Inc, South San Francisco, California 94080, USA

*These authors contributed equally

Correspondence

Nicholas J. Agard (agardn@gene.com)

and Felipe de Sousa e Melo (desousaf@gene.com)

Key words Cell-surface ligases, Antibody degrader, Targeted protein degradation

Figure 1e

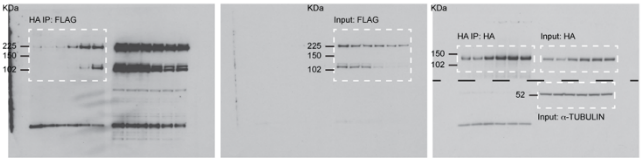

Figure 1h

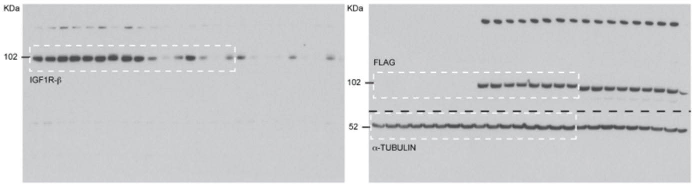

Figure 1i

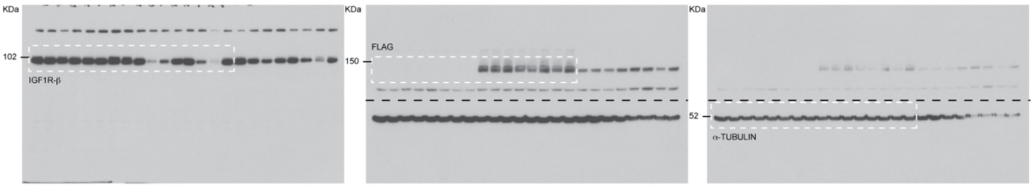

Figure 2f

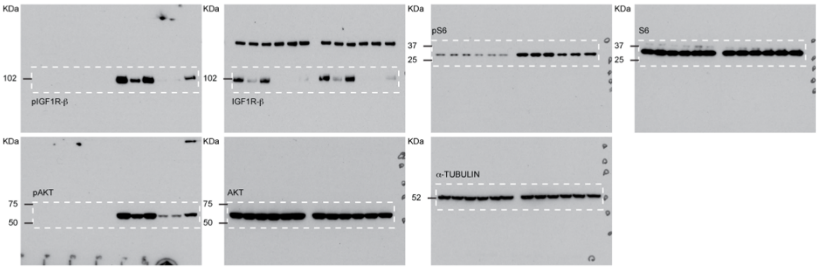

Figure 2i

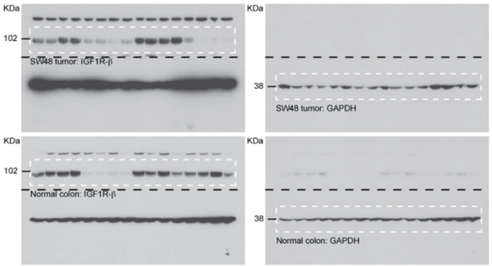

Figure 2j

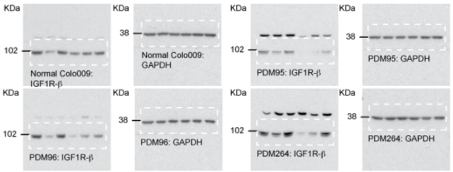

Figure 3c

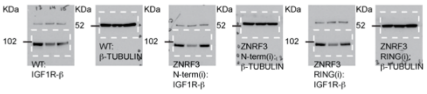

Figure 3d

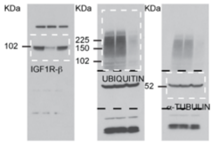

Figure 3e

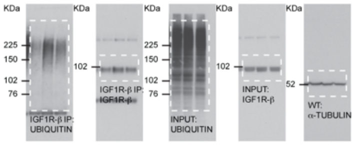

Figure 3f

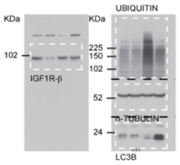

Figure 4a

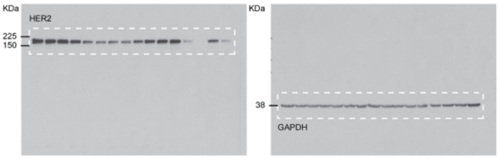

Figure 4b

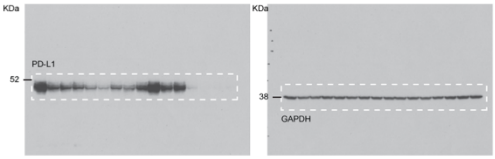

Figure 4d

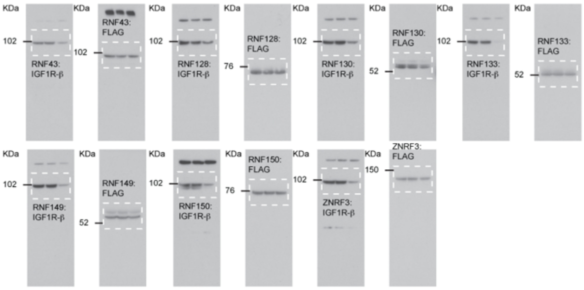

Figure 4j

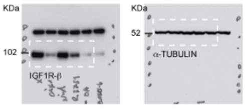

Supplementary Figure 1: Uncropped blots for western blot panels presented in main figures

Uncropped blots for western blot panels presented in main figures are outlined. Figure panels are indicated above the corresponding western blot set. Black dashed lines indicate where membranes were cut in the cases where multiple antibodies were probed simultaneously. Loading controls and the respective experimental samples were run on the same blots.

Extended Data figure 1e

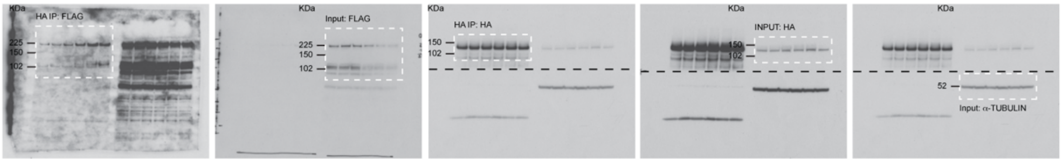

Extended Data figure 2f

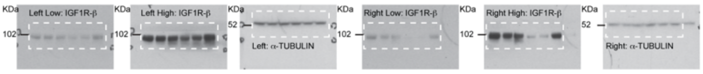

Extended Data figure 2j

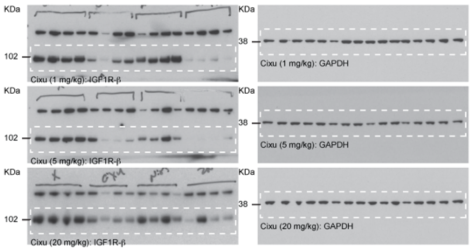

Extended Data figure 2g

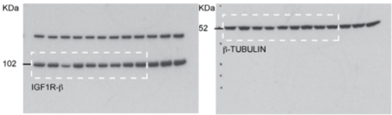

Extended Data figure 2i

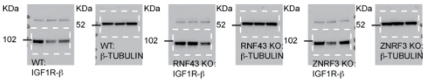

Extended Data figure 3e

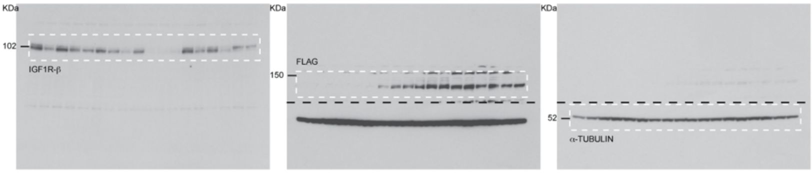

Extended Data figure 3f

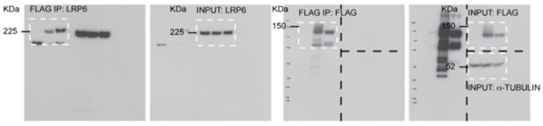

Extended Data figure 3g

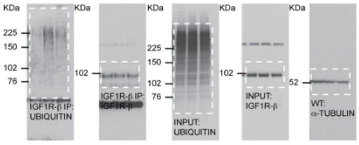

Extended Data figure 4a

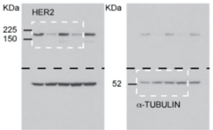

Extended Data figure 4b

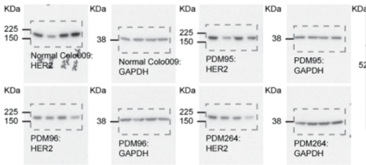

Extended Data figure 4c

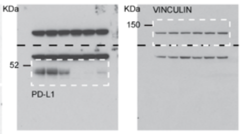

Extended Data figure 4d

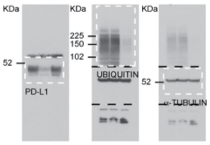

Extended Data figure 4e

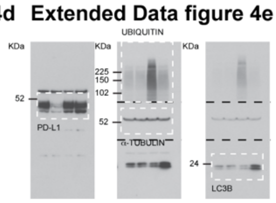

Extended Data figure 5c

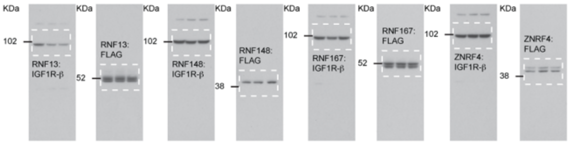

Extended Data figure 5d

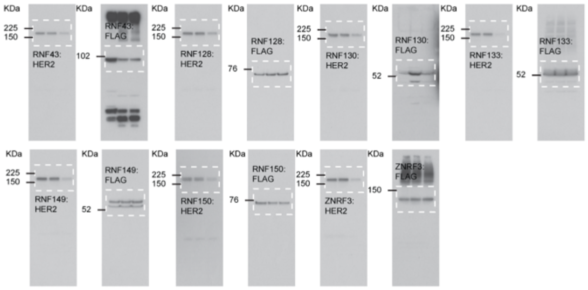

Extended Data figure 5e

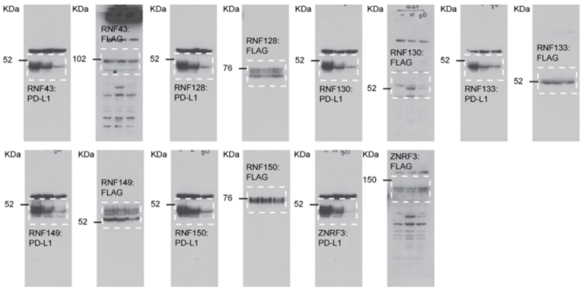

Supplementary Figure 2: Uncropped blots for western blot panels presented in Extended Data figures

Uncropped blots for western blot panels presented in Extended Data figures are outlined. Figure panels are indicated above the corresponding western blot set. Black dashed lines indicate where membranes were cut in the cases where multiple antibodies were probed simultaneously. Loading controls and the respective experimental samples were run on the same blots.

Gating strategy for Figure 1g

HT29 gD-ligases-FLAG

1. FSC SSC scatter gate

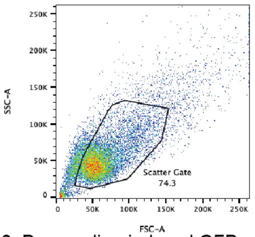

2. SSC: Single cell gate

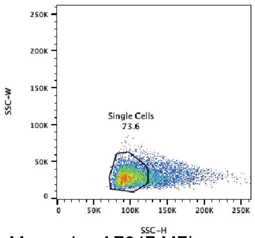

3. Doxycycline-induced GFP+ expression gate

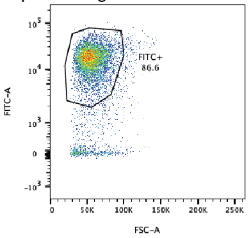

4. Measuring AF647 MFI

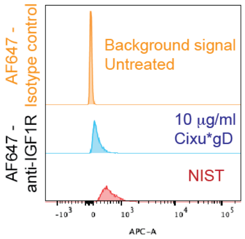

Gating strategy for Figure 3b and Extended Data figure 2h

HT29

1. FSC SSC scatter gate FSC-A x SSC-A
2. FSC: Single cell gate FSC-W x FSC-H
3. SSC: Single cell gate SSC-W x SSC-H

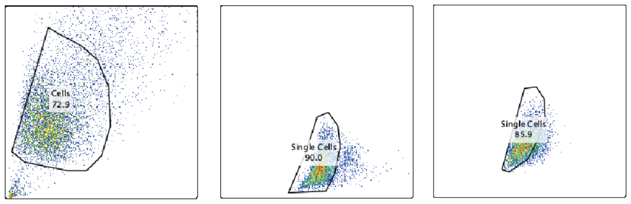

4. Measuring PE MFI

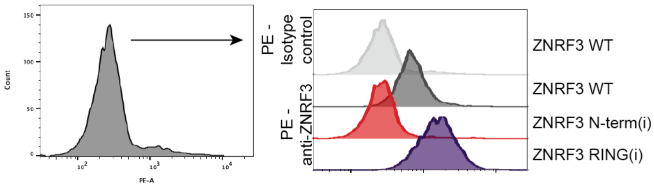

Gating strategy for Figure 4c

HT29 gD-RNF149-FLAG

1. FSC SSC scatter gate
2. SSC: Single cell gate
3. FSC: Single cell gate
4. Viability dye eFluor 405 Pacific Blue: Live cell gate
5. Measuring AF647 MFI

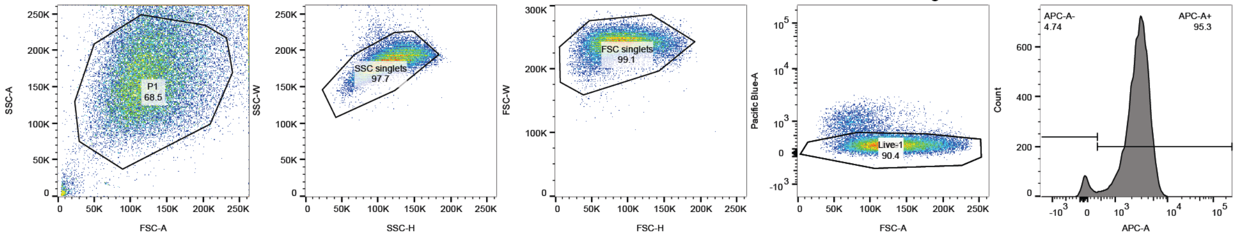

HT29 gD-ligases-FLAG

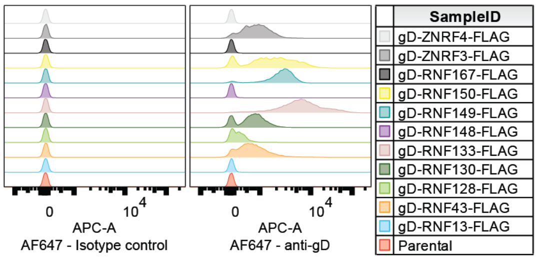

Gating strategy for Figure 4e

ASPC1 gD-RNF149-FLAG

1. FSC SSC scatter gate
2. Viability dye eFluor 780 BV786: Live cell gate
3. FSC: Single cell gate
4. SSC: Single cell gate
5. Doxycycline-induced GFP+ expression gate

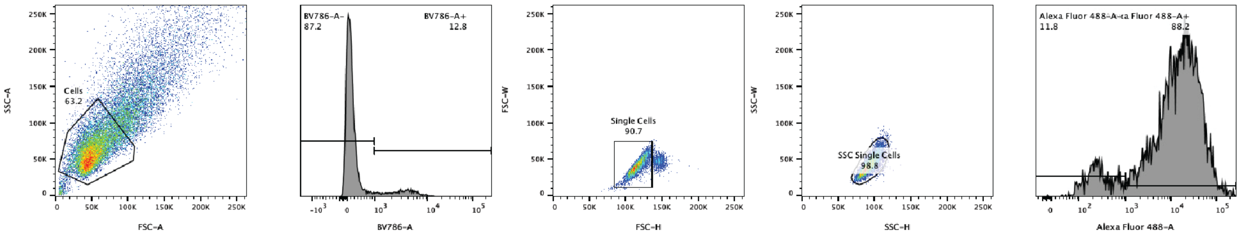

6. Measuring AF647 MFI

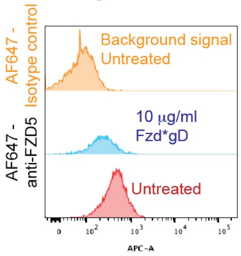

Supplementary Figure 3: Gating strategies for flow cytometry analysis

Gating strategies for flow cytometry analysis presented in the manuscript are outlined. Figure panels are indicated above the corresponding gating strategy.
