# Supplementary material for: Antibody targeting of E3 ubiquitin ligases for receptor degradation
Source: Nature. 2022 Sep 21;610(7930):182–9. doi: 10.1038/s41586-022-05235-6 (PMC9534761; doi:10.1038/s41586-022-05235-6)
Supplement: Supplementary file 2 — Reporting Summary [file 41586_2022_5235_MOESM2_ESM.pdf]

nature portfolio

Corresponding author(s): Nicholas J. Agard, Felipe de Sousa e Melo

Last updated by author(s): Aug 1, 2022

Reporting Summary

Nature Portfolio wishes to improve the reproducibility of the work that we publish. This form provides structure for consistency and transparency in reporting. For further information on Nature Portfolio policies, see our Editorial Policies and the Editorial Policy Checklist.

Statistics

For all statistical analyses, confirm that the following items are present in the figure legend, table legend, main text, or Methods section.

n/a	Confirmed	
☐	☒	The exact sample size (n) for each experimental group/condition, given as a discrete number and unit of measurement
☐	☒	A statement on whether measurements were taken from distinct samples or whether the same sample was measured repeatedly
☐	☒	The statistical test(s) used AND whether they are one- or two-sided<br>*Only common tests should be described solely by name; describe more complex techniques in the Methods section.*
☒	☐	A description of all covariates tested
☒	☐	A description of any assumptions or corrections, such as tests of normality and adjustment for multiple comparisons
☐	☒	A full description of the statistical parameters including central tendency (e.g. means) or other basic estimates (e.g. regression coefficient) AND variation (e.g. standard deviation) or associated estimates of uncertainty (e.g. confidence intervals)
☐	☒	For null hypothesis testing, the test statistic (e.g. F, t, r) with confidence intervals, effect sizes, degrees of freedom and P value noted<br>*Give P values as exact values whenever suitable.*
☒	☐	For Bayesian analysis, information on the choice of priors and Markov chain Monte Carlo settings
☒	☐	For hierarchical and complex designs, identification of the appropriate level for tests and full reporting of outcomes
☒	☐	Estimates of effect sizes (e.g. Cohen's d, Pearson's r), indicating how they were calculated

Our web collection on statistics for biologists contains articles on many of the points above.

Software and code

Policy information about availability of computer code

Data collection	RNA-sequencing (RNA-seq) libraries were sequenced on Illumina HiSeq 2500 sequencers<br>Flow cytometry was performed on a BD FACSymphony A3 Cell Analyzer and using the BD Diva acquisition software (version 9.0)<br>Immunohistochemistry pictures were acquired with an NIKON A1R using N3 elements software.
Data analysis	FlowJo 10.7.1 for flow cytometry analysis<br>Prismv8 and Prismv9<br>R (v4.1.0)<br>hg38 and mm9 gene models from Genentech's RefSeq-derived internal database IGIS (v4.0)<br>seqinr (v4.2)<br>Biostrings (v2.60.2)<br>hgu133plus2.db (v3.13.0)<br>annotationTools (v1.66.0)<br>Limma (v3.48.3)<br>SummarizedExperiment (v1.22.0)<br>IRanges (v2.26.0)<br>org.Hs.eg.db (v3.13.0)<br>SignalP (v5.0)<br>DeepLoc (v1.0)<br>nVennR (v0.2.3)<br>ggplot2 (v3.3.5)

plotly (v4.9.4.1)
msigdbr (v7.4.1) enrichplot (v1.12.2) clusterProfiler (v4.0.5) pheatmap (v1.0.12) protr (v1.6-2) circlize (v0.4.13) dendextend (v1.15.1)

All commercial and custom codes used to generate data presented in this study are available DOI: 10.5281/zenodo.6855630

For manuscripts utilizing custom algorithms or software that are central to the research but not yet described in published literature, software must be made available to editors and reviewers. We strongly encourage code deposition in a community repository (e.g. GitHub). See the Nature Portfolio guidelines for submitting code & software for further information.

Data

Policy information about availability of data

All manuscripts must include a data availability statement. This statement should provide the following information, where applicable:

- Accession codes, unique identifiers, or web links for publicly available datasets
- A description of any restrictions on data availability
- For clinical datasets or third party data, please ensure that the statement adheres to our policy

Epithelial Systems Biology Laboratory (ESBL) Human E3 Ubiquitin Ligases (accessed 6/2/2021); https://esbl.nhlbi.nih.gov/Databases/KSBP2/Targets/Lists/E3-ligases/
Genentech Release v6.0.2 of the GTEx project data (http://www.gtexportal.org/). This data currently contains 9120 RNA-Seq samples from 549 different donors.
Quantitative mass spectrometry proteomics data (raw data, metadata for experimental design, quantification results, and testing result) have been deposited to MassIVE (https://massive.ucsd.edu/ProteoSAFe/static/massive.jsp) with the data set identifier MSV000089542. Login credentials: Username: MSV000089542_reviewer. Password: znrf3
RNA seq data is available under GSE208372.
All source data is provided or accessible upon request.

Human research participants

Policy information about studies involving human research participants and Sex and Gender in Research.

Reporting on sex and gender	*Use the terms sex (biological attribute) and gender (shaped by social and cultural circumstances) carefully in order to avoid confusing both terms. Indicate if findings apply to only one sex or gender; describe whether sex and gender were considered in study design whether sex and/or gender was determined based on self-reporting or assigned and methods used. Provide in the source data disaggregated sex and gender data where this information has been collected, and consent has been obtained for sharing of individual-level data; provide overall numbers in this Reporting Summary. Please state if this information has not been collected. Report sex- and gender-based analyses where performed, justify reasons for lack of sex- and gender-based analysis.*
Population characteristics	*Describe the covariate-relevant population characteristics of the human research participants (e.g. age, genotypic information, past and current diagnosis and treatment categories). If you filled out the behavioural & social sciences study design questions and have nothing to add here, write "See above."*
Recruitment	*Describe how participants were recruited. Outline any potential self-selection bias or other biases that may be present and how these are likely to impact results.*
Ethics oversight	*Identify the organization(s) that approved the study protocol.*

Note that full information on the approval of the study protocol must also be provided in the manuscript.

Field-specific reporting

Please select the one below that is the best fit for your research. If you are not sure, read the appropriate sections before making your selection.

☒ Life sciences ☐ Behavioural & social sciences ☐ Ecological, evolutionary & environmental sciences

For a reference copy of the document with all sections, see nature.com/documents/nr-reporting-summary-flat.pdf

Life sciences study design

All studies must disclose on these points even when the disclosure is negative.

Sample size	Statistical methods were not used to predetermine sample size. Sample size followed common standard of n=2 or more biological replicates. The number of animals used for each experiments was estimated based on the variability in tumor take and tumor growth observed for each models in previous in house studies. Number of animals are described in relevant figure legends or relevant figures and were all equal or above n=4 per group.
Data exclusions	No data was excluded from the manuscript. For Extended Data Fig. 1f outlier value excluded from graph is provided in corresponding source file.

Replication	All experiments presented are representative of at least two biological repeats that showed reproducible results unless otherwise indicated. Exceptions include the high throughput binding analysis of RNF43 and ZNRF3 bivalent antibodies generated through the rabbit and rat antibody campaigns presented in Fig. 2 and Extended Data Fig. 2. Due to the large number of campaign antibodies, these were produced at small scale, which precluded biological repeats. For these, all antibody binding was orthogonally validated through flow cytometry with two independent biological experiments. Copy number analysis presented in Extended Fig. 3a and 3b represent technical replicates of one biological experiment. The low copy number of these receptors is at the lower limit of detection that can only be detected with high efficiency in 125-I labeling of the antibody, which varies between assays. To minimize batch effect, the entire experiment across all cell lines and receptors was performed in single batch.
Randomization	For animal studies animals were randomized after tumors had reached a size of ~ 400 mm3 to receive a single intraperitoneal injection of the PROTABs. No other experiments were randomized. To counter for potential batch effect, all in vitro treatment were performed on the same plate.
Blinding	Blinding during experimental procedures were not required because experimental conditions could be easily identified from the data itself.

Reporting for specific materials, systems and methods

We require information from authors about some types of materials, experimental systems and methods used in many studies. Here, indicate whether each material, system or method listed is relevant to your study. If you are not sure if a list item applies to your research, read the appropriate section before selecting a response.

Materials & experimental systems

n/a	Involved in the study	
☐	☒	Antibodies
☐	☒	Eukaryotic cell lines
☒	☐	Palaeontology and archaeology
☐	☒	Animals and other organisms
☒	☐	Clinical data
☒	☐	Dual use research of concern

Methods

n/a	Involved in the study	
☒	☐	ChIP-seq
☐	☒	Flow cytometry
☒	☐	MRI-based neuroimaging

Antibodies

Antibodies used

Akt (pan) (40D4) Mouse mAb; Cell Signaling; 2920S; Lot 8; Dilution 1:1,000
Anti-HA High Affinity (3F10); Roche Diagnostics; 11867423001; Lot 45715900; Dilution 1:1,000
Anti-mouse IgG, HRP-linked Antibody; Cell Signaling; 7076S; Lot 36; Dilution 1:5,000
Anti-rabbit IgG, HRP-linked Antibody; Cell Signaling; 7074S; Lot 30; Dilution 1:5,000
Anti-rat IgG, HRP-linked Antibody; Cell Signaling; 7077S; Lot 14; Dilution 1:5,000
GAPDH (14C10) Rabbit mAb (HRP Conjugate); Cell Signaling; 3683; Lot 4; Dilution 1:1,000
Goat anti-Human IgG (H+L) Secondary Antibody, Alexa Fluor 647; Invitrogen; A21445; Lot 2339821; Dilution 1:1,000
Goat anti-Mouse IgG (H+L) Secondary Antibody, HRP; Invitrogen; 32430; Lot VD301382; Dilution 1:5000
Goat anti-Rabbit IgG (H+L) Secondary Antibody, HRP; Invitrogen; 32460; Lot VE30198; Dilution 1:5000
HER2/ErbB2 (D8F12) XP® Rabbit mAb; Cell Signaling; 4290S; Lot 6; Dilution 1:1,000
IGF-I Receptor β (D23H3) XP®Rabbit mAb; Cell Signaling; 9750S; Lot 5-7; Dilution:1,000
IGF1R (1H7), APC; eBioscience; 17-8849-42; Lot 2330467; Dilution 1:20
IRDye® 800CW Donkey anti-Mouse IgG Secondary Antibody; LI-COR; 926-32212; Lot D10414-15 and D00930-09; Dilution 1,5000
IRDye® 800CW Goat anti-Rabbit IgG Secondary Antibody; LI-COR; 926-32211; Lot D10629-12 and D01110-10; Dilution 1,5000
LC3B antibody; Novus Biologicals; NB100-2220; Lot EU/EU-3; Dilution 1:1,000
LIVE/DEAD™ Fixable Violet Dead Cell Stain Kit, for 405 nm excitation; Thermo Fisher; L34964; Lot 2208471; Dilution 1,000
LRP6 (C5C7) Rabbit mAb; Cell Signaling; 2560S; Lot 11; Dilution 1:1,000
Monoclonal ANTI-FLAG® M2 antibody produced in mouse; Sigma-Aldrich; F3165; Lot SLCG2330 and SLBT6752; Dilution 1:1,000
Monoclonal Anti-α-Tubulin antibody produced in mouse; Sigma-Aldrich; T9026; Lot 099M4773V; Dilution 1:10,000
PD-L1 (E1L3N®) XP® Rabbit mAb; Cell Signaling; 13684S; Lot 18; Dilution 1:1,000
Phospho-Akt (Ser473) Antibody; Cell Signaling; 9271S; Lot 15; Dilution 1:1,000
Phospho-IGF-I Receptor β (Tyr1135/1136)/Insulin Receptor β (Tyr1150/1151) (19H7) Rabbit mAb; Cell Signaling; 3024S; Lot 11; Dilution 1:1,000
Phospho-S6 Ribosomal Protein (Ser235/236) Antibody; Cell Signaling; 2211S; Lot 23; Dilution 1:1,000
Polyclonal Rabbit Anti-Human c-erbB-2 Oncoprotein; Agilent (DAKO); A0485; Lot 20083958; Dilution 1:1,000
S6 Ribosomal Protein (5G10) Rabbit mAb; Cell Signaling; 2217S; Lot 10; Dilution 1:1,000
Ubiquitin monoclonal antibody (P4G7-H11); Enzo; ADI-SPA-203-F; Lot 04062139; Dilution 1:1,000
Ultra-LEAF™ Purified Human IgG1 Isotype Control Recombinant Antibody; BioLegend; 403502; Lot B322065; Dilution 10 ug/ml
Vinculin Antibody; Cell Signaling; 4650S; Lot 5; Dilution 1:1,000
β-Tubulin (9F3) Rabbit mAb; Cell Signaling; 2128L; Lot 11; Dilution 1:1,000

Validation

All commercial antibodies utilized in this study were selected based on provided manufacturer's validation data. For in house antibodies, such as RNF43, ZNRF3 and anti-gD used for bispecific antibody generation, WB and flow cytometry antibodies were validated in house by ELISA, SPR, dot blots (data not shown) as well as WB (data not shown) and flow cytometry (Extended Data Fig. 2

and Fig. 3 for representative RNF43 and ZNRF3 in house bivalent antibodies; Fig. 4 for anti-gD antibody) using cell lines overexpressing the proteins of interest and in the case of RNF43 and ZNRF3 KO cells were also used for flow cytometry validation.

Eukaryotic cell lines

Policy information about cell lines and Sex and Gender in Research

Cell line source(s)	HEK293T, LS1034, KM12, DLD1, HT115, LS180, LS513, SW1417, HT55, GP2D, RKO, COLO678, SW48 and ASPC1 lines and primary human organoids were obtained from ATCC and maintained by Cell Central, an in house cell line repository, at Genentech, Inc. Primary murine organoid cultures were also used (described in methods under "organoids" subsection).
Authentication	All lines are authenticated using standard genotyping methods.
Mycoplasma contamination	All cell lines acquired from Cell Central are routinely tested for mycoplasma and no mycoplasma contamination was reported for parental cell lines utilized. Genetically engineered lines were generated using parental lines obtained from cell central and were not further tested for mycoplasma contamination.
Commonly misidentified lines (See ICLAC register)	No misidentified lines were used in this study.

Animals and other research organisms

Policy information about studies involving animals; ARRIVE guidelines recommended for reporting animal research, and Sex and Gender in Research

Laboratory animals	WT B57BL/6 mice (000664) and NOD.Cg-PrkdcscidIl2rgtm1Wjl/SzJ (NSG) (colony 005557) mice were purchased from the Jackson Laboratory. Sprague Dawley rats were obtained from Charles River, Hollister, CA. White rabbits were obtained from WORC (Western Oregon Rabbit Co). Females of 6 to 12 weeks old were used for experiments. Standard housing conditions, including dark/light cycle, ambient temperature and humidity were used.
Wild animals	The study did not involve wild animals.
Reporting on sex	*Indicate if findings apply to only one sex; describe whether sex was considered in study design, methods used for assigning sex. Provide data disaggregated for sex where this information has been collected in the source data as appropriate; provide overall numbers in this Reporting Summary. Please state if this information has not been collected. Report sex-based analyses where performed, justify reasons for lack of sex-based analysis.*
Field-collected samples	The study did not involve samples collected from the field.
Ethics oversight	Animal studies were approved by Genentech's Institutional Animal Care and Use Committee and adhere to the NRC Guidelines for the Care and Use of Laboratory Animals.

Note that full information on the approval of the study protocol must also be provided in the manuscript.

Flow Cytometry

Plots

Confirm that:

☒ The axis labels state the marker and fluorochrome used (e.g. CD4-FITC).

☒ The axis scales are clearly visible. Include numbers along axes only for bottom left plot of group (a 'group' is an analysis of identical markers).

☒ All plots are contour plots with outliers or pseudocolor plots.

☒ A numerical value for number of cells or percentage (with statistics) is provided.

Methodology

Sample preparation	96-well plates cell surface staining detailed protocol:<br>• Day1:<br>o Plate cells in 96-well round bottom plate at 250,000 cells/well (adjust depending on cell line used)<br>• Day2:<br>o Perform media exchange (+ dox at 1 ug/ml if using inducible cell line)<br>• Day3:<br>o After 48 hours of cell plating and 24 hours of dox induction (if applicable) prepare cells for FACS staining<br>o Aspirate media using gel loading tips attached to 8-well aspirator (to avoid cell suction and loss)<br>o Wash cells in 100 µl/well PBS<br>o Aspirate PBS using gel loading tips attached to 8-well aspirator (to avoid cell suction and loss)<br>o Detach cells using 5 mM EDTA (this gives a better signal for cell surface staining compared to trypsin and accutase)<br>o Incubate for 15 minutes at 37 °C (adjust depending on cell line used)

o Examine cells under microscope to ensure that they are fully detached and pipet cells up and down to facilitate the process
o Add 100 µl/well media to detached cells, mix by pipetting and spin at 1,200 rpm for 5 minutes at 4°C
o Aspirate media using gel loading tips attached to 8-well aspirator (to avoid cell suction and loss)
o Resuspend cell pellets in 100 µl/well FACS buffer and block by incubating for 10 minutes on ice
o After incubation, spin at 1,200 rpm for 5 minutes at 4°C
o Aspirate media using gel loading tips attached to 8-well aspirator (to avoid cell suction and loss)
o Resuspend cell pellets in 100 µl/well 1ry antibody solution (prepared in FACS buffer) and incubate for 1 hour on ice
o After incubation, spin at 1,200 rpm for 5 minutes at 4°C
o Aspirate media using gel loading tips attached to 8-well aspirator (to avoid cell suction and loss)
o Resuspend cell pellets in 100 µl/well FACS buffer and spin at 1,200 rpm for 5 minutes at 4°C
o Repeat wash step a total of 3 times
o Resuspend cell pellets in 100 µl/well 2ry antibody solution (prepared in FACS buffer) and incubate for 1 hour on ice covered in foil
o After incubation, spin at 1,200 rpm for 5 minutes at 4°C
o Aspirate media using gel loading tips attached to 8-well aspirator (to avoid cell suction and loss)
o Resuspend cell pellets in 100 µl/well FACS buffer and spin at 1,200 rpm for 5 minutes at 4°C
o Repeat wash step a total of 2 times
o Resuspend cell pellets in 100 µl/well LIVE/DEAD fixable dead cell stain solution (prepared in FACS buffer or PBS) and incubate for 30 minutes on ice covered in foil
o After incubation, spin at 1,200 rpm for 5 minutes at 4°C
o Aspirate media using gel loading tips attached to 8-well aspirator (to avoid cell suction and loss)
o Resuspend cell pellets in 100 µl/well FACS buffer and spin at 1,200 rpm for 5 minutes at 4°C
o Resuspend cell pellets in 100 µl/well fixative solution and incubate for 15 minutes at RT covered in foil
o After incubation, spin at 1,200 rpm for 5 minutes at 4°C
o Aspirate media using gel loading tips attached to 8-well aspirator (to avoid cell suction and loss)
o Resuspend cell pellets in 100 µl/well FACS buffer and spin at 1,200 rpm for 5 minutes at 4°C
o Repeat wash step a total of 2 times
o Resuspend cell pellets in 100 µl/well FACS buffer
o Analyze cells immediately using flow cytometry or store covered in foil at 4°C for later processing
Primary antibodies
Anti-Human IGF-1R-APC, Clone 1H7, Invitrogen/eBioscience, Catalog # 17-8849-42, Lot 2172983
Ultra-LEAF purified human IgG1 isotype control; Biolegend; 403502
hRNF43.RNF43-129HC.hIgG1; In house; Req ID: 547062; PUR ID: 612194
hZNRF3.ZNRF3-275HC.hIgG1; In house; Req ID: 547073; PUR ID: 612064

Secondary antibody
Goat anti-Human (H+L) cross-absorbed 2ry antibody, Alexa Fluor 647; Invitrogen; A21445
Goat anti-human IgG (H+L) 2ry antibody, PE; Invitrogen; PA1-86078

Live/dead dye
LIVE/DEAD fixable violet dead cell stain, for 405 nm excitation; Thermo Fisher Scientific; L34964A

Fixative solution
Image-iT fixative solution (4 % formaldehyde, methanol free);; Thermo Fisher Scientific; FB002

Instrument

BD FACSCelestaTM Flow Cytometer #660344

Software

BD FACSDivaTM for collection and FlowJo 10.7.1 for analysis.

Cell population abundance

Flow cytometry was mainly used to analyze cell-surface staining of various proteins

Gating strategy

For endogenous IGF1R cell-surface staining presented throughout the manuscript and exogenous ligase cell-surface staining presented in Fig.4 cell gating was performed as follows: FSC-A/SSC-A manual scatter gate on cell population followed by FSC-H/FSC-W and SSC-H/SSC-W manual gate on single cells. Live cells were selected in applicable experiments. Detailed gating strategies used are outlined in Supplementary Figure 3.

☒ Tick this box to confirm that a figure exemplifying the gating strategy is provided in the Supplementary Information.
